# Activation of the Antiviral Kinase PKR and Viral Countermeasures

**DOI:** 10.3390/v1030523

**Published:** 2009-10-27

**Authors:** Bianca Dauber, Thorsten Wolff

**Affiliations:** 1 Department of Medical Microbiology & Immunology, University of Alberta, 632 Heritage Medical Research Center, Edmonton, AB, T6G 2S2, Canada; 2 P15, Robert Koch-Institute/Nordufer 20, 13353 Berlin, Germany

**Keywords:** PKR, virus, dsRNA, innate immunity, immune evasion

## Abstract

The interferon-induced double-stranded (ds)RNA-dependent protein kinase (PKR) limits viral replication by an eIF2α-mediated block of translation. Although many negative-strand RNA viruses activate PKR, the responsible RNAs have long remained elusive, as dsRNA, the canonical activator of PKR, has not been detected in cells infected with such viruses. In this review we focus on the activating RNA molecules of different virus families, in particular the negative-strand RNA viruses. We discuss the recently identified non-canonical activators 5′-triphosphate RNA and the vRNP of influenza virus and give an update on strategies of selected RNA and DNA viruses to prevent activation of PKR.

## 1. Introduction

The presence and replication of viral nucleic acids in vertebrate cells triggers innate immune reactions, in particular the induction of type I interferon (IFN) genes and the activation of antiviral enzymes [1]. The double-stranded (ds) RNA-dependent protein kinase (PKR) is a key executor of this antiviral response along with other interferon-stimulated gene products such as the 2′,5′-oligoadenylate synthetases and the Mx proteins [2]. PKR is present in non-stimulated cells at basal levels that vary in regard to the tissue type and the degree of differentiation [3]. However, its expression level is upregulated by type I IFN, which allows a robust response to viral infections [4]. Human PKR is a latent serine/threonine kinase of 551 amino acids with two consecutive *N*-terminal dsRNA-binding motifs, a linker domain, and a *C*-terminal kinase domain [5]. Activation of PKR during viral infection is mediated by recognition of viral nucleic acids, which induces a structural rearrangement and brings two PKR monomers into close proximity [6]. This allows back-to-back dimerization with the kinase domain facing outwards, and the concomitant autophosphorylation of the critical threonine residues 446 and 451 in the activation loop [7–9]. Active PKR can then bind and phosphorylate its best studied natural target, the serine residue 51 of the alpha subunit of the eukaryotic translation initiation factor 2 (eIF2α) [10–12]. In mammalian cells, eIF2-GTP delivers Met-tRNAi to the 40S ribosome. After GTP hydrolysis eIF2-GDP is released and regenerated to eIF2-GTP by the GTP-exchange factor eIF2B. The increased affinity of phosphorylated eIF2α for eIF2B leads to sequestration of this rate limiting factor and results in inhibition of translation initiation [13]. As viruses critically depend on the cellular translation machinery, the PKR mediated block of translation strongly impairs efficient viral reproduction and spread. In addition, PKR can act as signal transducer in the IκB/NFκB pathway and plays a role in the control of cellular processes such as apoptosis, cell growth and differentiation, and response to cellular stresses other than viral infection [14]. Accordingly, PKR can also be activated by the polyanionic molecule heparin [15] or the protein activator PACT [16]. Under non-stress conditions activation of PKR by PACT is prevented by interaction of PACT with TRBP (TAR RNA binding protein) [17].

PKR and the 2′,5′-oligoadenylate synthetases (OAS) were the first cellular proteins identified that respond to dsRNA produced during viral infection [18]. As such they can be referred to as pattern recognition receptors (PRRs), a term first used to account for recognition of pathogen associated molecular patterns (PAMPs) by Toll-like receptors (TLRs) and RIG-I-like receptors (RLRs) that initiate synthesis of type I interferons (IFN-α and IFN-β) [19,20]. Defining the molecular structure of viral PAMPs has extended our understanding of how PRRs discriminate between viral and ‘self’ RNA. Such work has, for instance, added endosomal guanosine- or uridine-rich ssRNA (activators of TLR7/TLR8) and intracellular RNA carrying a 5′-triphosphate group (activating RIG-I) to the list of viral PAMPs [19,21]. Considering that PKR is able to recognize viral RNA from a broad range of virus families, it is not surprising that the repertoire of PKR activating RNA molecules goes beyond the canonical dsRNA.

In the following section we summarize the activating RNA molecules of different virus families, in particular the non-canonical activators established in recent studies such as 5′-triphosphate RNAs and vRNPs of influenza virus. We also give an update on the diverse viral strategies that prevent activation of PKR.

## 2. Activation of PKR by Viral Ribonucleotides

**Figure 1.**
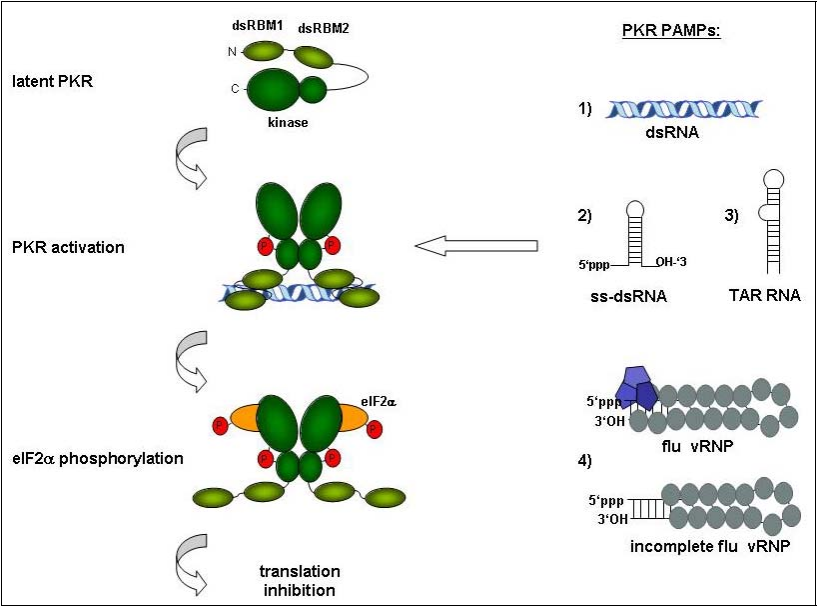
Activation of PKR by different viral and synthetic RNAs. Latent PKR binds to (1) perfect dsRNA of viral or synthetic origin, (2) synthetic structured RNA with single stranded tails and a 5′triphosphate, (3) HIV-1 TAR RNA or (4) (possibly incomplete) influenza virus vRNP. This leads to dimerization and autophosphorylation of PKR. Active PKR then phosphorylates its substrate eIF2α, which results in a block of translation. PKR consists of two dsRNA-binding motifs (dsRBM1 + dsRBM2 in light green) and the *N*-terminal and *C*-terminal lobe of the kinase domain (dark green).

Historically, PKR was identified as the protein kinase responsible for translation inhibition in response to synthetic dsRNA polyinosinic acid: polycytidylic acid (poly(I:C)) or viral dsRNA derived from vaccinia virus or poliovirus infected cells [18]. Actual binding of PKR to dsRNA was first shown by partial purification of PKR using poly(I:C)-Sepharose [22]. Later studies concluded that PKR requires a minimum of 33bp of perfect dsRNA for induction and around 80 bp and longer for optimal activation [23]. Binding of dsRNA shorter than 33 bp did not activate PKR, but rather inhibited activation by long dsRNA [23]. However, experimental data have accumulated indicating that the range of nucleic acids capable to activate PKR is considerably broader than was originally appreciated (Figure 1).

### 2.1. Synthetic RNAs

In order to characterize the structural features that comprise a PKR PAMP, many laboratories have for practical reasons used synthetic RNAs such as poly(I:C) instead of purified viral RNAs. Poly(I:C) is easy to work with; however, the stretches of dsRNA are heterogeneous in length and inosine is a relatively rare ribonucleotide in cells or viruses [24]. Other sources are chemically synthesised RNAs or RNAs transcribed by the RNA polymerases of the bacteriophages T7, T3 or SP6. These RNAs allow a precise analysis of the impact of length, structure and ribonucleotide modifications on PKR activation. One has to keep in mind though, that *in vitro* transcribed RNAs contain a triphosphate group at their 5′end. Most cellular RNAs lack a 5′-triphosphate as it is processed to a 7-methyl guanosine cap for mRNA or monophosphate for tRNA and rRNA [25]. As many bacterial and viral RNAs contain a 5′-triphosphate group, this structure can serve as a marker of non-self RNA. Nallagatla and coworkers have shown that long duplex RNA, the canonical PKR activator, does not depend on 5′-triphosphates. However, this modification is critical for the activation of PKR by shorter RNAs with a 16 bp stem loop and 10 to 15 nt single-stranded tails as well as by a 47nt ssRNA with minimal secondary structure, *i.e.*, two short stem-loops of 5 and 4 bp [26]. Another interesting finding is that internal nucleoside modifications, which are quite common in cellular RNAs, reduce the ability of RNAs to serve as PKR PAMPs, providing another means of discrimination between self and non-self RNAs [27].

In addition to PKR, intracellular RNA is also detected by RIG-I, a major sensor protein facilitating type I IFN upregulation in response to several virus families [21]. There are similarities and differences regarding the nature of the RNA detected by either protein. Whereas RIG-I mediated induction of IFN-α/β genes is believed to depend on RNAs carrying a 5′triphosphate [28,29], the requirement for a 5′-triphosphate group to activate PKR seems to depend on the structure of the RNA as discussed earlier [26]. Another difference between RIG-I and PKR is that RIG-I is able to detect 5′-triphosphate RNAs as short as 19 nt, whereas the minimal length for detection by PKR appears to be 30 bp for dsRNA or ∼47 nt for ss-dsRNA [23,26,29]. Initial studies indicating that RIG-I, in contrast to PKR, is activated by single-stranded RNA have been challenged by findings that stress the requirement for base-paired stretches in addition to the 5′-triphosphate [30–32]. These studies also add another caveat regarding the use of *in vitro* transcripts: T7 polymerase possesses a RNA-dependent RNA polymerase activity that can result in non-templated hairpin RNAs as byproducts of the transcription reaction, necessitating purification by denaturing polyacrylamide gel electrophoresis to isolate the intended RNA products [31–34].

### 2.2. Viral RNAs

Considering the diverse genome structures and replication strategies of different virus families, it is conceivable that the PAMPs generated during viral infection are similarly diverse. However, there are obstacles that complicate the identification of potential PKR activating RNAs within virus-infected cells. For one, most viruses express proteins or RNAs that inhibit PKR or its downstream effects (see below). This necessitates the use of naturally occurring virus mutants or recombinant viruses that lack this activity. The detection and/or isolation of the respective PAMPs pose another challenge. Although dsRNA can be detected by indirect immunofluorescence microscopy, the most widely used antibody only detects dsRNAs >40bp [35]. Furthermore, RNAs extracted from infected cells are not complexed with proteins, as they would have been in the cell, and could possibly fold into a different secondary structure, which might alter their activity as PAMPs. Despite these difficulties, progress has been made in defining viral PAMPs recognized by PKR. We give an overview of what is known so far about the origin and structure of these RNA molecules.

Complex DNA viruses such as vaccinia virus, adenovirus, herpes simplex virus or cytomegalovirus transcribe open reading frames in opposite orientations, which can lead to formation of long duplex RNAs. Evidence for these viral dsRNA came from experiments performing extraction and re-annealing of RNA from virus infected cells [36–38] and detection by indirect immunofluorescence microscopy using an antibody that detects dsRNA >40bp [39–41]. The latter method also confirmed the presence of dsRNA in cells infected with reovirus which has a dsRNA genome [42] and virus families with a positive-strand RNA genome such as *Togaviridae* (rubella virus, Semliki Forest virus, Sindbis virus [43,44]), *Coronaviridae* (SARS corona virus [39], *Picornaviridae* (encephalomyocarditis virus [39]), and *Flaviviridae* (Kunjin virus, poliovirus, tick-borne encephalitis virus, hepatitis C virus, dengue virus [45–48]). In the case of positive-strand RNA viruses the dsRNA might represent replication intermediates or long stretches of extensively base-paired secondary structure elements [49]. There is conflicting data regarding the IRES element of hepatitis C virus which has been suggested to either inhibit [50] or activate PKR [51]. Another well-studied RNA with secondary structure is the human immunodeficiency virus type 1 (HIV-1) trans-activation responsive (TAR) element at the 5′-termini of HIV-1 mRNAs. The TAR RNA consists of a stem-loop interrupted by three bulges and has been shown to bind to and activate PKR [51–55]. *In vitro* experiments suggest that dimerization of TAR RNA is necessary to provide a dsRNA molecule of sufficient length to engage two PKR molecules and induce dimerization and activation of PKR [56]. This mode of activation might be similar to the activation of PKR by self RNAs that contain highly structured regions, such as the mRNAs of IFN-γ and α-tropomyosin; a mechanism that is used to regulate expression of these mRNAs [57,58].

For negative sense ssRNA viruses it has long been thought that dsRNAs activating cellular receptors represent replication intermediates [59]. For the families of the *Orthomyxo*- and *Bornaviridae* such a scenario is unlikely, since production of viral RNAs with opposite polarities is a nuclear event [60], whereas activation of PKR is believed to occur in the cytoplasm [61]. Although dsRNA has been extracted from influenza A virus infected cells [59], this might have been a result of the extraction procedure during which both cytoplasmic and nuclear ribonucleoproteins (RNPs) of opposite polarity are stripped of their nucleoproteins and, hence, can easily form long duplex RNAs. Indeed, a dsRNA-specific antibody did not detect significant levels of dsRNA in cells infected with the influenza A wild-type virus or a deletion virus (ΔNS1) that lacks the PKR inhibitor NS1 [28,39]. It is possible that PKR is activated by PACT, which would not require dsRNA [16,39,62]. However, a recent study addressing the role of PACT in viral infection by using PACT knockout mice neither detected an alteration of dsRNA induced phosphorylation of eIF2α nor any influence on replication of the negative-strand RNA viruses VSV and Sendai virus [63].

The availability of reverse genetic procedures has not only allowed the identification of viral inhibitors of PKR but also enabled a closer examination of the viral PAMPs of PKR in particular for influenza viruses. Recombinant influenza A and B viruses with a mutated or deleted gene for the nonstructural protein 1 (NS1) are strong activators of PKR [64–66] and of RIG-I controlled IFN-α/β genes [67–72]. The first piece of evidence that defined the nature of the PKR PAMP in influenza infection came from studies by Hatada *et al*. who showed that PKR can be activated *in vitro* by purified influenza A virion RNA and a model vRNA [66]. Both RNA species carry a 5′-triphosphate group and engage in base-pairing between partially complementary 5′-terminal and 3′-terminal ends. As described above, these features comprise PKR PAMPs [26]. Furthermore, RNA isolated from influenza virions activated RIG-I in a 5′-triphosphate dependent manner [28]. However, in infected cells vRNAs are complexed with nucleoprotein and the viral polymerase proteins forming ribonucleoproteins (vRNP) [60]. This raises the question whether the danger signals are concealed in such nucleoprotein complexes or whether vRNP is itself an activating ligand of PKR. Analysis of an influenza B virus expressing a non-functional NS1 protein provided the first evidence that influenza virus RNP indeed functions as a non-canonical activator of PKR in the cytosol [55]. The study showed that (i) PKR autophosphorylation in infected cells occurred concomitantly with the cytosolic appearance of vRNP, when a functional NS1 protein was absent, (ii) PKR activation was largely abolished when the nucleo-cytoplasmic export of vRNP was blocked by LMB treatment, and (iii) purified vRNP activated PKR in an *in vitro* kinase assay in a dsRNA-dependent manner. Mechanistically, the base-pairing between the 14–16 nt at the vRNA termini, which form a panhandle and/or a related corkscrew structure [73–75], could provide the dsRNA structure for activating PKR. This terminal structure may not be covered in all the vRNP complexes produced during virus infection. It was not possible to answer the dependency on the 5′-triphosphate conclusively, as the phosphatase used would not only hydrolyze the 5′-triphosphate, but would also remove the phosphate groups on activated PKR and ATP, and elimination of the phosphatase by phenol extraction would destroy the RNP structure. However, phosphatase treatment strongly reduced activation of PKR by synthetic influenza virus model vRNA containing the terminal 5′- and 3′- ends, indicating a contribution of the 5′-triphosphate to this process [55]. This study is the first to show that a natural viral RNA/RNP with a 5′-triphosphate group can trigger PKR activation.

The findings described above raise the question whether other negative strand RNA viruses activate PKR in a similar way. One observation that favours this hypothesis is that the conserved ends of the genomic RNAs of several members of the *Bunyaviridae* also form a structured panhandle [76] and the level of phosphorylated eIF2α increased during infection with the prototypic Bunyamwera virus and the Rift Valley fever virus lacking the PKR inhibitor NSs [77–79]. Non-segmented negative-strand RNA viruses also activate PKR; for instance, the Ebola virus VP35 protein counteracts stimulation of PKR, indicating indirectly that members of the *Filoviridae* family can activate and thus try to inhibit activation of the kinase [80,81]. In addition, the replication and virulence of vesicular stomatitis virus, a member of the *Rhabdoviridae*, is strongly enhanced in PKR-deficient mice [82]. Interestingly, most genomic RNAs of single-stranded RNA viruses carry a 5′-triphosphate group. This was shown for Zaire Ebola virus (*Filoviridae*), Nipah virus and measles virus (*Paramyxoviridae*), Lassa virus (*Arenaviridae*), Rift Valley fever virus (*Bunyaviridae*), rabies virus and vesicular stomatitis virus (*Rhabdoviridae*) [29,83] and suggests that their vRNPs are potential PKR PAMPs. Some viruses such as Hantaan virus, Crimean Congo hemorrhagic fever virus (*Bunyaviridae*) and Borna disease virus carry a 5′-monophosphate, possibly to escape detection by host cell innate immune mechanisms [83]. It has to be taken into account though, that the genomic RNA of members of the order *Mononegavirales* is more tightly encapsidated by the nucleoprotein than the genomic RNA of influenza viruses [84–86]. Therefore, more experimental work is needed to determine the potential of vRNPs of negative-strand viruses other than influenza virus to trigger PKR activation.

Another RNA that has been considered to be a potential PAMP is the 5′triphosphate containing leader RNA that is transcribed from the most promoter-proximal gene of the *Paramyxo*-, *Rhabdo*- and *Filoviridae* genome [87,88]. The leader RNA is not encapsidated until substantial amounts of nucleoprotein have accumulated and it has been implicated in activating RIG-I mediated IFN induction by measles virus [89]. However, work by Bitko *et al*., indicated that the leader RNA of the respiratory syncycial virus and Sendai virus is shielded from RIG-I in a complex with the cellular La protein [90]. No studies have been conducted so far to test whether leader RNA might activate PKR.

Finally, some members of the *Paramyxoviridae* actually produce long duplex RNAs under conditions where synthesis of genomic and antigenomic RNA is not tightly regulated. Takeuchi *et al.* showed that, in contrast to wild-type Sendai virus infected cells, significant amounts of dsRNA were detected by indirect immunofluorescence microscopy in cells infected with a mutant virus expressing an inactive C protein [91]. Their findings indicate that the C protein prevents excessive RNA synthesis, thus preventing the production of dsRNA and activation of PKR. Similarly, infection with a measles virus lacking the C protein led to activation of PKR and phosphorylation of eIF2α. The *C*-deficient virus showed a restricted growth phenotype, which was partially restored by depletion of PKR, whereas the PKR status had no impact on wild-type virus replication [92]. Although the dsRNA content in cells infected with the C knockout virus has not been analysed, the measles virus C protein has been implicated in regulating RNA synthesis as well [93]. Gainey *et al*. showed a similar strategy for the SV5 virus, although in this case the P/V proteins are instrumental in limiting activation of PKR [94]. In case of the Newcastle disease virus, the Ulster strain has been shown to produce dsRNA in amounts detectable by the dsRNA antibody and induced phosphorylation of PKR and eIF2α [91]. Interestingly, wild-type measles and Sendai viruses cannot prevent PKR activation by the Vaccinia virus ΔE3L, that lacks the PKR inhibitor E3L, or the Newcastle disease virus (Ulster strain), respectively [91,92]. These observations suggest that at least some paramyxoviruses do not require a PKR inhibitor, as they tightly regulate the replication process and thus keep viral RNA at a level that can be complexed by nucleocapsid proteins and masked from PKR.

## 3. Viral Countermeasures

Many virus families have evolved gene products targeting PKR, illustrating the importance of this kinase to antiviral defense. The inhibitory mechanisms are manifold and include sequestration of viral dsRNA by a viral protein, prevention of PKR activation through direct interaction with viral proteins or viral decoy RNA, regulation of eIF2α phosphorylation through a viral pseudosubstrate or recruitment of a cellular phosphatase, or PKR degradation (summarized in [95–98]). Here, we focus on new mechanistic aspects of PKR inhibition by the influenza virus NS1 protein and additional PKR inhibitory proteins expressed by Rift Valley Fever virus, Ebola virus and cytomegalovirus, which were described only recently (Table 2).

### 3.1. Influenza A and B viruses

It is well known that the influenza A and B virus NS1 proteins (A/NS1 and B/NS1, respectively) function as PKR antagonists since mutant viruses with defects in the NS1 gene, but not wild-type virus, are potent PKR activators [64–66]. This inhibition of PKR is critical for virus production, as mutant viruses with *loss-of-inhibition* mutations in the NS1 gene are severely attenuated in *PKR^+/^*^+^ but not in *PKR^–/–^* mice and embryonic fibroblasts [55,64]. The NS1 proteins of both virus types are multifunctional proteins consisting of 202–237 and 281 amino acids (aa), respectively. Both NS1 proteins also downregulate the RIG-I mediated activation of type I IFN genes [28,65,67,68,70,71]. For the A/NS1 protein this activity was recently shown to involve inhibition of TRIM25-mediated RIG-I ubiqitination [99]. The A/NS1 protein was also shown to inhibit the maturation and export of cellular pre-mRNAs, to enhance translation, to inhibit the 2′,5′-oligoadenylate synthetase (OAS) and to activate the phosphatidylinositol 3-kinase (PI3K) [100–108] (summarized in [109]). In contrast, it is a specific function of the influenza B virus NS1 protein to inhibit the conjugation of the antiviral ISG15 gene product to cellular targets [110] and to modify the nuclear speckle compartment [111]. Although the overall sequence identity is below 25%, both NS1 proteins carry a similarly structured *N*-terminal dsRNA binding domain located at positions 1–73 (type A) and 1–93 (type B) [112]. Both NS1 proteins bind to the same RNAs *in vitro* including synthetic dsRNA, U6 RNA, and poly(A)-RNA [113] and a model vRNA with base-paired 3′-terminal and 5′-terminal ends [114].

The longstanding hypothesis has been that NS1 proteins prevent PKR activation by sequestering dsRNA. However, recent data indicate that this model needs to be revised. Work in our laboratory showed that the influenza B virus NS1 protein and PKR form an immunoprecipitable complex in infected cells that was sensitive to treatment with dsRNA-specific RNase and required a functional NS1 dsRNA-binding domain [55]. As described in Section 2.2, influenza vRNPs provide the major stimulus for PKR activation, possibly through the partially base-paired region at their vRNA termini. Interestingly, vRNA was detected in PKR-NS1 immunoprecipitates from infected cell lysate [55]. This raises the possibility that B/NS1 protein blocks activation of PKR by cytosolic vRNP through the formation of a heterotrimeric complex. It cannot be ruled out, though, that binding of B/NS1 to PKR in infected cells is mediated by a yet undetermined viral or host-derived nucleic acid. However, this study established that the key activity of the B/NS1 dsRNA binding domain is to silence PKR, as virulence and virus production of virus mutants expressing dsRNA-binding deficient NS1 proteins were restored to wild-type levels in *PKR*^–/–^ mice and fibroblasts [55].

For the influenza A virus NS1 protein there is inconsistent data concerning the mode of PKR inhibition. Early studies suggested an important role of the A/NS1 protein’s dsRNA-binding activity. DsRNA binding-deficient NS1 mutant protein of the A/PR/8/34 strain did not inhibit PKR activation and eIF2α phosphorylation by dsRNA and model vRNAs in *in vitro* assays [66,115]. Furthermore, A/Udorn/72 viruses expressing NS1 proteins (K62N or A132T) that are dsRNA binding-deficient as part of their *ts* phenotype activated PKR in infected cells at the non-permissive temperature [66]. However, a later study concluded that dsRNA-binding by the NS1 protein is not essential for inhibition of PKR [116]. Cells infected with a recombinant A/Udorn/72 virus expressing a dsRNA binding-deficient NS1 protein (NS1-R38A) did not show phosphorylation of PKR and the NS1-R38A mutant protein also inhibited PKR activation by dsRNA and PACT *in vitro* [116]. Instead, the inhibition of PKR was shown to correlate with an interaction of NS1 and PKR that was abolished by mutation of the NS1 residues 123/124 or 126/127 [117]. Additionally, work by Tan & Katze demonstrated that the residues 7 to 48 located in the dsRNA-binding domain of the A/Udorn/72 NS1 protein are essential for the PKR interaction [118]. Taken together, these results indicate that both the dsRNA-binding domain and the region between amino acid residues 123 and 127 contribute to this interaction. Regarding the role of the influenza A virus NS1 protein in counteracting the antiviral response, it was suggested that the main function of the dsRNA-binding domain was to target the antiviral 2′-5′-OAS/RNaseL system. Another strategy to inhibit PKR during influenza virus infection has been thought to involve activation of the cellular protein p58^IPK^ [119]. P58^IPK^ is activated at the post-transcriptional level, interacts with PKR and reduces PKR-mediated eIF2α phosphorylation, thereby increasing viral mRNA translation [119,120]. Although such an activity is expected to support viral replication on the cellular level, a recent study revealed that gene knockout of p58^IPK^ leads to increased lung pathology, immune cell apoptosis, PKR activation and mortality in influenza A virus infected mice [121]. Therefore, activation of p58^IPK^ may rather be seen as a control mechanism to limit an excessive PKR response and prolong host survival than as a strategy of influenza virus to inhibit PKR.

### 3.2. Rift Valley fever virus

Recent studies suggested that the NSs protein of Rift Valley fever virus (RVFV), a member of the *Bunyaviridae* genus *Phlebovirus*, induces the proteasome-mediated degradation of PKR in order to escape from this antiviral response [77,78]. PKR was active and eIF2α was phosphorylated in cells infected with an RVFV lacking functional NSs [77,78]. Interestingly, eIF2α phosphorylation was greatly enhanced by actinomycin D or α-amanitin treatment that was used to mimic the transcriptional inhibition of cellular genes conferred by the NSs protein [78]. Furthermore, it has been shown that it is an autonomous function of the NSs protein to induce PKR degradation [78]. Experiments in *PKR*^–/–^ mice and fibroblasts confirmed that the kinase plays a major role in limiting virus production and pathogenicity of RVFVs lacking the NSs gene or expressing a non-functional truncated NSs protein [77,78]. However, in the experiments performed by Habjan *et al*. PKR deficiency could not completely ablate the antiviral effect of IFN and the NSs-deficient virus killed PKR–/– mice with slower kinetics than wt mice, indicating that other antiviral proteins such as the Mx protein and the OAS/RNaseL pathway help inhibit RVFV replication [77]. The NSs protein also prevents transcriptional induction of the type I interferon genes, an activity exerted by the NSs protein of all members of the *Bunyaviridae* tested so far [122–126]. In contrast, degradation of PKR seems to be unique to RVFV as a recombinant RVFV expressing NSs protein of the sandfly fever Sicilian virus (genus *Phlebovirus*) or the LaCrosse virus (genus *Orthobunyavirus*) lacked this activity [77]. Furthermore, PKR is activated by the Bunyamwera virus (genus *Orthobunyavirus*), and the virus is moderately sensitive to the antiviral action of PKR, regardless of the presence or absence of NSs [79]. Hence, the RVFV-specific capacity to block the PKR antiviral response likely contributes to the high pathogenicity of RVFV. Generation of recombinant RVFV expressing mutant NSs protein will help to determine the NSs domains responsible for the degradation of PKR and define the role of cellular proteins in this process. Poliovirus is the only other virus known to induce degradation of PKR [127]. Although poliovirus RNA and proteins are required, cellular rather than the viral proteases seem to be involved in the degradation [128]. However, the precise mechanism has not been determined yet.

### 3.3. Ebola virus

The Ebola virus VP35 protein serves as an antagonist of PKR activation [80,81]. Furthermore, VP35 abrogates type I IFN induction by inhibiting activation of IRF3 and IRF7 through inhibition of the IRF3 kinases TBK1/IKKε and modulation of the sumoylation machinery [129–132] and plays an essential role as a polymerase cofactor and a structural component in virus assembly [133,134]. VP35 contains an *N*-terminal coiled-coil domain required for its oligomerization [135] and a *C*-terminal dsRNA-binding region [136,137]. Oligomerization is critical for VP35 activity as mutation or deletion of the coiled-coil domain destroyed the capacity to inhibit PKR and prevent IRF3 activation [80,135]. The latter activity was restored by providing a heterologous oligomerization domain [135]. DsRNA-binding is facilitated by basic amino acid residues R305, K309 and R312 in the C-terminal IRF3 inhibitory domain, which is required for the IFN-antagonist activity of VP35 [136,137]. Thus, a recombinant Ebola virus expressing dsRNA binding-deficient VP35 with the mutation R312A was a strong inducer of the innate immune response and was severely attenuated in mice [138,139]. On the contrary, mutation of more than one of the basic residues R305, K309 and R312 was needed to abrogate VP35’s PKR antagonistic activity, indicating that dsRNA-binding is not essential for this function [80,81]. This raised the question whether VP35 directly interacts with PKR. However, pull-down experiments failed to detect such an interaction [80]. Taken together, these results indicate that the mechanism of PKR inhibition does not depend on RNA-binding or direct interaction with PKR. Instead, the requirement for the IRF3-inhibitory domain of VP35 points to a possible involvement of cellular proteins that contribute to the inhibition of PKR. Generation of a recombinant Ebola virus expressing a VP35 with both R312A and K309A mutations would certainly aid the identification of these potential cellular interaction partners or signaling events.

### 3.4. Cytomegalovirus

Human cytomegalovirus (HCMV) encodes two related proteins, pTRS1 and pIRS1, which bind dsRNA and block activation of PKR [140–142]. Neither one of the proteins by itself is essential for viral replication, indicating a functional redundancy, although deletion of pTRS1 results in a modest decrease in viral titers probably due to its role in virus assembly [143,144]. However, deletion of both genes in HCMV[ΔI/ΔT] resulted in a severe replication defect, inhibition of cellular and viral protein synthesis and phosphorylation of eIF2α [41]. In contrast to PKR, the OAS/RNaseL pathway is not activated in HCMV[ΔI/ΔT] infected cells [41], although pIRS1 and pTRS1 have been shown to prevent activation of RNaseL in cells infected with a Vaccinia virus that lacks the E3L protein (VVΔE3L), an inhibitor of PKR and OAS/RNaseL [141]. Mechanistically, both the *N*-terminal non-canonical dsRNA-binding domain and the C-terminal region of pTRS1 and pIRS1 are necessary for counteracting the antiviral action of PKR [142,145]. Interestingly, both pTRS1 and pIRS1 interact with PKR and this requires the C-terminus of either protein [145]. Whether dsRNA contributes to this interaction has not yet been established. Hakki *et al*. further showed that PKR accumulates in the nucleus of HCMV infected cells and in VVΔE3L infected cells coexpressing pTRS1, indicating that this is an autonomous function of pTRS1 [145]. It has to be noted, though, that in HCMV infection PKR is not totally depleted from the cytoplasm, in contrast to the VVΔE3L-infected, pTRS1 expressing cells. Taken together, the results suggest that the interaction of pTRS1 and pIRS1 with dsRNA and PKR prevents activation of PKR at least in part by confining PKR to the nucleus away from its activator, dsRNA, and from its target, eIF2α.

The murine cytomegalovirus (MCMV) *m142* and *m143* genes belong to the same gene family as the HCMV genes *IRS1* and *TRS1* [146,147]. The m142 and m143 proteins also act as PKR inhibitors, but, in contrast to pTRS1/pIRS1, both proteins are needed for inhibition. Deletion of either protein results in PKR and eIF2α phosphorylation, translational shutdown and decreased replication [148]. Furthermore, replication of an m142/m142-deficient MCMV was restored in *PKR*^–/–^ MEFs, but not in *RNaseL*^–/–^ cells, confirming PKR as the main target of m142/m143 [40]. Consistent with this result, neither MCMV nor the m142/m143 deletion virus activated the OAS/RNaseL pathway [40]. In the course of determining the mechanism of PKR inhibition, both proteins were shown to interact with one another forming a heterotetramer and to function together to bind dsRNA [149,150]. Both proteins together also interacted with PKR independent of dsRNA [40,149]. As described for HMCV, PKR accumulated in the nucleus and also in insoluble fractions in the cytoplasm of infected cells, suggesting that this relocalization might sequester PKR to compartments where it cannot exert its antiviral action [149]. It will be interesting to assess the impact of dsRNA-binding and protein interaction on relocalization of PKR and how this relocalization relates to the PKR antagonist activity of m142/m142.

## 4. Conclusions

Many RNA and DNA virus families have acquired one or more gene product(s) that reduce the induction of the latent kinase PKR or phosphorylation of its substrate eIF2α by very diverse mechanisms. Research in the past decade has revealed that the capacity to antagonize this cellular defence is an important aspect of the virulence and/or host specificity of these viral pathogens. There is recent evidence for rapid evolution of *PKR* genes in primates and positive selection at specific amino acid sites, supporting the view that this kinase evolves under the constant pressure of antagonistic viral gene functions [151,152]. Although a lot of information has accumulated on PKR, a number of questions remain concerning the biology of this conserved kinase. These include the precise sequence of events leading from the latent, monomeric, form to the fully phosphorylated dimer, whether allelic differences in *PKR* are associated with different susceptibility to certain viruses, and the precise roles of several of its cellular interaction partners. A particular technical challenge concerns the characterization of structures within natural viral nucleic acids, which trigger PKR activation inside cells. Clearly, research directed to close these gaps in our knowledge will provide valuable insights into the ongoing arms race between viral pathogens and their hosts.

## Figures and Tables

**Table 1.** Viral RNA structures that potentially activate PKR.

**Virus (genome)**	**dsRNA detected in IFA**	**Origin of PKR activating RNA**	**Reference**
VacV, AdV, HSV-1, HCMV, MCMV (DNA)	+	overlapping converging transcription	[36–41]
HIV-1 (RNA/DNA)	n. a.	TAR RNA/possibly as dimer	[52–54,56]
ReoV (dsRNA)	+	dsRNA genome	[39,42,49]
Rubella V., SFV, SINV, SARS CoV, EMCV, Kunjin V., PolioV, TBEV, HCV, DENV. (+ssRNA)	+	replication intermediates or base-paired secondary structure elements	[39,43–48]
Influenza V. (-ssRNA, segm.)	–	vRNP/complementary 3′ and 5′ termini of vRNA (flu B virus)	[28,39,55]
LaCrosse V. (-ssRNA, segm.)	–	panhandle structure of vRNA?	[39,76]
SenV (-ssRNA, non-segm.)	–	?	[91]
SenV C protein mutant, NDV Ulster strain (-ssRNA, non-segm.)	+	replication intermediates	[91]

**Table 2.** Discussed viral gene products that inhibit PKR activation.

**Viral product**	**Virus**	**Mode of inhibition**
NS1	influenza A virus	direct interaction with PKR
NS1	influenza B virus	dsRNA-mediated interaction with PKR
NSs	Rift Valley Fever virus	proteasome-mediated degradation of PKR
VP35	Ebola Virus	unknown
pTRS1/pIRS1	human CMV	relocalization of PKR, interaction with PKR
m142/m143	murine CMV	relocalization of PKR, direct interaction with PKR
